# The Foreign Journals: Medical Jurisprudence and Toxicology

**Published:** 1841-07

**Authors:** 


### MEDICAL JURISPRUDENCE AND TOXICOLOGY.

### *On Asphyxia from Carbonic Acid.* By M. Devergie.

In the 11th Number of this Journal, at p. 241, our readers will find the report of a singular case of murder by carbonic acid, recorded by M. Devergie. Some of the medico-legal questions which were raised in that case were of so novel a character, that the author has been induced to reexamine the whole subject. We shall here give an abstract of the results of his observations and experiments.

1. *On the circumstances under which asphyxia by carbonic acid may take place.*

It is commonly supposed that asphyxia by carbonic acid is not likely to occur in those cases, where the source of combustion does not exist in the apartment itself in which an individual may be found apparently dead. This is an error, for it is well known that when two chimneys or the flues of stoves communicate, if a fire be lighted in one room, the vapour may be drawn through the communicating chimney by a downward current into a distant room. The distribution of the vapours of burning fuel will therefore depend greatly upon the state of expansion or contraction in the air of such communicating apartments. This source of asphyxia has been hitherto overlooked. M. D'Arcet has published several cases which show that serious accidents may thus occur without the cause being even suspected by the medical attendant.

2. *What takes place in an apartment in which a charcoal fire has been lighted?*

When the charcoal is kindled, and so long as it burns it absorbs oxygen from the air, producing thereby carbonic acid and carburetted hydrogen: the latter, however, is converted as soon as it is formed into carbonic acid and water. Since the carbonic acid thus produced occupies the same bulk as the oxygen which was required for its production, it follows that the quantity of air in the apartment can undergo no augmentation, if we except the addition to it of a very small portion of carburetted hydrogen that may escape combustion. But, although there is no actual increase in quantity, there is a vast difference of bulk, which is due to the great expansion caused in gases by a rise of temperature. The heat of combustion is such, as probably to give a threefold volume to the air of an apartment; and to cause the upper and lower strata rapidly to change places. This increased bulk must continue during the whole period of combustion; and the dilated air will endeavour to find an outlet through every fissure or opening connected with the apartment. If there be any impediment to this escape, it will exert a pressure in proportion to its state of expansion on all surrounding bodies: a pressure which would materially impede respiration, and accelerate the access of asphyxia.

There is another fact of medico-legal interest. While the combustion lasts, there are two currents of air circulating through the room; the one ascending of heated air, and therefore rich in carbonic acid, the other descending of cold air. It is by means of the ascending current that the carbonic acid produced in combustion is equally diffused through all parts of the chamber. To establish this point, several experiments were made. Among others two vessels for collecting gases were opened in the apartment, so soon as the combustion had terminated: the one being placed at the top, and the other on the floor. The quantity of carbonic acid collected was perceptibly the same in the two cases, the proportion being about one fiftieth part. *Hence it follows that during the combustion of charcoal, the carbonic acid is equally diffused throughout the space in which it is produced.*

The air of such an apartment is unfit for respiration: 1st, because it is deficient in the quantity of oxygen, necessary for the maintenance of that function; and 2d, because it is contaminated by deleterious gases.

So soon as the combustion is finished, there is a tendency to the production of an equilibrium in temperature: the expanded air slowly contracts and occupies less bulk. If any air had previously escaped from the apartment through openings, a fresh portion now enters to supply its place, so that when the equilibrium is perfect, the air is less vitiated than it was during combustion. If the apartment were so close that no air could escape, then the pressure of the expanded mass diminishes with the decrease of temperature, and resumes its original condition; but in this case, it is just as much contaminated as it was while the charcoal was burning.

3. *Do the gases formed separate according to their specific gravities after the air has cooled?*

This is a question of material importance in relation to asphyxia, and to illustrate its application, the following supposed case may be taken: Two persons are found asphyxiated in the same chamber; the one may be stretched on the bed, the other lying on the floor. Which of these two has been more powerfully affected by the asphyxiating cause, (the charcoal vapour)? The solution of this question must rest partly on reasoning and partly on experience. After the complete combustion of charcoal, the respirable air becomes specifically lighter: a large quantity of oxygen has been removed from it in the production of carbonic acid; nitrogen now predominates, and this gas is lighter than air. Hence we may affirm that irrespective of the carbonic acid produced, the specific gravity of the air of the chamber has diminished. The specific gravity of carbonic acid is one half greater than that of air (1·5:1); but in consequence of the change which the air in the chamber has undergone, the difference becomes still greater. We might therefore infer that when once the air has cooled, or even during the act of cooling, the carbonic acid would tend to fall to the lowest part of the chamber. To this inference may be opposed the experiments of Dalton on the diffusion of gases, which establish that these bodies, notwithstanding the difference in their specific gravities, have a tendency to mix uniformly on contact. M. Devergie was, however, induced to think that under the above-mentioned circumstances, carbonic acid would collect on the floor of an apartment, in a stratum of variable density; and to remove any doubt on the point, the following experiment was performed:

Two vessels proper for collecting gas were placed the one at the highest and the other at the lowest part of a moderately large chamber, in which a quantity of charcoal was burnt. The next day, when the apartment had become thoroughly cooled, the bottles of gas were removed: a quantity of solution of potash was put into each, and they were immediately closed. Two hours afterwards, the solution was placed in a proper vessel connected with a graduated jar, and sulphuric acid added until the potash was saturated. The liquid in the bottle from the floor of the chamber yielded 150 parts of carbonic acid, while the other vessel, which was at the summit of the apartment, gave only 32 parts. A cat placed on the floor uttered painful cries for an hour and a half, and the next morning was found dead.

We must therefore consider it as proved by this experiment, that after combustion has ceased, and while the apartment is cooling, the carbonic acid *separates from the air by its greater specific gravity,* and forms on the floor a stratum, of which the thickness must vary according to the quantity of fuel consumed. Still the gas does not separate entirely: a portion remains mixed with the air in the upper part of the room, which contains about $\frac{1}{78}$th; while that on the floor contains $\frac{1}{9}$th. It is possible that this phenomenon may be due to gaseous mixtures, taking place in definite proportions, as well as to the effect of the difference of temperature in the different strata of air. The fact is somewhat analogous to the separation of metals, according to their density, in melting certain kinds of allays. The following case is quoted as confirmatory of this view:

A gentleman tried to destroy himself by burning charcoal in his chamber. For this purpose he lighted a brazier and threw himself on the bed. Not finding that he became affected by the vapour, he for the time abandoned his design. Upon mentioning the circumstance to some friends, as if another party had been concerned, he learned from a professional man present that carbonic acid only produced serious effects to those placed on the floor of a chamber. A few days afterwards, this gentleman was found dead in his room, sitting at the foot of his bed, and a candle still burning on a table. It was evident that he had destroyed himself by charcoal vapour.

4. *On the nature of asphyxiating media.*

The only data which exist on this subject are derived from the experiments of Orfila. The analysis of the vapour, produced from charcoal, according to this chemist, varied with the degree of combustion; when this was *slow*, the carbonic acid formed about one seventh of the mixture; when *rapid*, one ninth: these results appear so paradoxical, that M. Devergie places but little reliance on them.

Charcoal vapour, when existing in large quantity, has a bluish tint owing to the smoke mixed with it. This colour the author has found to disappear in twelve hours after the completion of combustion, and he thinks it may become still more rapidly dissipated. This fact, he observes, may be useful as a datum to determine the period at which asphyxia probably commenced in an unknown case. The vapour has likewise a peculiarly disagreeable odour, producing nausea and cephalalgia: it is chiefly perceptible when the charcoal is first ignited, and disappears so soon as the combustion has become very active. It probably has some influence in the production of asphyxia, and to it may be attributed the narcotic effects of charcoal. This charcoal vapour is heavier than atmospheric air; it reddens litmus, gives a white precipitate with lime water, and extinguishes burning substances. In relation to the last character, it is necessary to observe, that in a state of admixture with air, it may produce asphyxia without extinguishing the flame of a lighted candle. Among the experiments performed, a cat and two birds were asphyxiated in an apartment, in which five candles were burning. This vapour never contains sufficient carbonic oxide or carburetted hydrogen to become inflamed by the approach of a candle.

5. *On the quantity of charcoal which is required to be burnt, in order to render a certain quantity of air deleterious.*

Varin showed that animals perished in about three minutes, when plunged into an atmosphere containing about one fifth of its volume of carbonic acid. But it is not necessary for asphyxia to ensue, that the atmosphere in a room, in which charcoal is burning, should acquire such an impregnation as this. There is obviously a great difference between a mixture of free carbonic acid and air, and a mixture containing carbonic acid actually produced at the expense of the oxygen of the air. Thus in the two cases, the air may not contain more than ten per cent. of carbonic acid; but where this has resulted from combustion, the atmosphere will contain seven per cent. less of oxygen and seven per cent. more of nitrogen than in the case where free carbonic acid has been mechanically added to air. On the other hand, Varin proved that the strongest proportion in which carbonic acid could be mixed with air without rendering it injurious to respiration is about two per cent. If only one fourth of the oxygen be removed by combustion, it will contaminate the air with one twentieth of carbonic acid, and render it asphyxiating.

Experiments were then performed to determine how much charcoal was required to be burnt, in order to remove one fourth of the oxygen from a given quantity of air. In these experiments it was necessary to allow for the bulk of the charcoal; the wood from which it was made; the degree to which the charring process had gone on; the quantity of hygrometric water present, and the different proportion of salts entering into its composition. We need not detail these experiments, since, as the author allows, from the circumstances above mentioned, they can only be regarded as remote approximations to the truth. An answer to a question of this kind must therefore be a matter of pure speculation.

6. *How to determine the capacity of a chamber in which asphyxia has taken place.*

This is rather a question for a surveyor. We are directed to multiply the length in feet by the width, and the product by the height, to obtain the measurement in cubic feet. The author lays great stress upon our making an allowance for any want of angularity in cupboards, closets, &c.

7. *How to determine the quantity of charcoal which has been consumed.*

Charcoal in burning leaves on the average about four per cent. or $\frac{1}{25}$th of its weight of ashes. All we have to do is to collect the ashes and to multiply them by 25. Here we are met by the objection, that different kinds of charcoal yield different quantities; and we can test the accuracy of our inference only in those cases where some of the charcoal used happens to remain unconsumed. The author advises us to satisfy ourselves that there were no ashes already in the brazier, prior to the asphyxiating attempt being made.

8. *On the closure of apartments and its influence in the production of asphyxia.*

It is a matter of general belief, that for asphyxia to take place it is necessary that the apartment in which the charcoal is burning should be perfectly closed. That this view is erroneous many cases on record clearly show. The perfect closure of a room is a circumstance highly favorable to the production of asphyxia; but it is not an indispensable condition. In the author's experiments the windows were loosely secured, and the chimney not perfectly closed. Persons have been repeatedly found asphyxiated in apartments, where there was a pretty free communication with the external air, by means either of a half-opened window or flue. We are then justified in affirming that this imperfect closure of outlets is unfavorable to the production of asphyxia, but that it does not render its occurrence impossible.

9. *On the influence of the situation of the person in the occurrence of asphyxia..*

A case has been already related (3), in which it was shown that the asphyxiating atmosphere had not the same intensity throughout. An individual, who escaped this form of death in a first attempt while lying on a bed, fell a victim to it when, on a second occasion, he placed himself on the floor. It has been proved, that during the actual combustion of charcoal, the air is equally impregnated with carbonic acid throughout; but that in the act of cooling this gas fell by its greater specific gravity to the lower part of the chamber. The following points may therefore be considered as settled: 1, That, whatever be the situation of a person, he will perish from asphyxia if the quantity of charcoal consumed was sufficient, *during its combustion*, to render the atmosphere deleterious: 2, If two persons are situated, the one on the floor and the other three or four feet above that level, the latter may not be asphyxiated while the former may perish, provided the quantity of charcoal consumed was insufficient to render the air deleterious during its combustion, but still sufficient to impregnate with carbonic acid during the act of cooling the lowest strata of air.

10. *On the influence of sex, age, and profession* (!), *on asphyxia.*

All persons are not equally susceptible of the influence of charcoal vapour. Those who consume much of this kind of fuel, for domestic or other purposes, resist its effects better than others. According to the Police Registers of 1834-5, there were in Paris 360 cases of asphyxia by carbonic acid. Out of this number there were nineteen cases of two persons together (men and women), and only one case of two men together. There were only three instances, out of these nineteen cases, in which one of the two persons could be resuscitated, and in each of these the subject was a female. The number of females saved is greater than of males. In 184 cases of asphyxia, occurring during the year 1835, there were eighteen females saved out of seventy-three cases, or about one fourth, and nineteen males out of eighty-three cases, or the proportion of about one fifth.

MM. Marye and Ollivier have quoted cases which lead to a contrary inference, showing that there is a greater power of resistance to the effects of this gas in males than in females. M. Devergie pronounces these to be isolated facts, and prefers his own results.

*Annales d'Hygiène. Jan.* 1840.

[Remarks.—Such is an abstract of M. Devergie's late researches on asphyxia from carbonic acid. Since we last adverted to this subject (No. XI. July 1838), one or two cases have occurred in this metropolis which have given rise to considerable discussion, and it cannot be concealed that there is even now so great a difference of opinion in the profession, that probably no two medical witnesses would agree in their answers to some of the questions proposed by M. Devergie. There are many statements made by the author of this paper, to the correctness of which we cannot assent; and as we should regret to be the means of disseminating views which might give rise to much mischief, if implicitly relied on, we shall here offer a few remarks on some of the most disputable doctrines.

In the second query we find it stated, that the air by expansion, if unable to find a ready outlet, will exert a compressing force on the body, impeding respiration, and accelerating asphyxia. We can understand that phenomena of this kind might ensue, if the man as well as the fuel were confined in a hermetically sealed vessel; but it seems to us impossible to admit, that in ordinary apartments, any such serious effects as the author imagines can take place.

In the third query, M. Devergie assumes from his experiments, that gases of different specific gravities mixed by heat, are capable of separating from each other to a greater or less extent on cooling, so that the heavier gas falls in great proportion on the soil or floor. To us this appears a material question in relation to death by carbonic acid; and the grounds on which such a statement is based require to be closely scrutinized.

The law of the diffusion of gases, now so well known to all chemists, has contributed to remove the old opinion, that carbonic acid from its weight had a tendency to collect and remain on the lowest levels. Gases are now known to mix readily on contact, whatever be their relative densities. Thus carbonic acid will rise into hydrogen, and hydrogen will fall into carbonic acid, although the difference of sp. gr. is as 22 : 1. There is no proof of this mixture of gases taking place in *definite* proportions: on the contrary, they are generally considered to mix in *all* proportions. Again, another fact commonly received by chemists is, that when once mixed, gases do not again separate from each other according to their specific gravities. M. Devergie, while he admits the principle of diffusion, affirms, that where this has been aided by heat, a partial separation takes place on cooling, so that carbonic acid and air, although mixed when heated, separate when cold. The single experiment which he adduces in support of his peculiar view is unsatisfactory. It would be impossible by such a method as that pursued by him to determine, with the least pretension to accuracy or certainty, the relative proportions of carbonic acid when mixed with air. This will be easily proved by trying the experiment with a mixture containing a measured quantity of pure carbonic acid. The case which he adduces is altogether inappropriate as an illustration. How are we to know that, in the first attempt at suicide, when the man was lying on the bed, the air of his apartment became sufficiently impregnated with carbonic acid to destroy him? yet it is here assumed, that in the two attempts there was an equally fatal contamination, and that the deceased only died in the second attempt, because he had placed himself on the floor!

We might stop here; but we are desirous of showing, not merely that M. Devergie's opinion is unsupported by his own experiments and reasoning, but that it is at variance with the most simple facts of almost daily occurrence. If on cooling, carbonic acid separated from air, as alleged by him, it is not to be expected that when the gases are once perfectly cold they would readily mix, nor that the heavy gas would again rise into the air and diffuse itself with a rapidity proportioned to the surface of contact, and yet that these phenomena do actually occur the most simple experiments will show. If a large glass vessel, having a diameter at the mouth of about five inches, with a depth of sixteen inches, be filled with carbonic acid and exposed to the access of air in a quiet and cool apartment, the whole of the gas will disappear from it in the course of a few hours. How is this to be accounted for if M. Devergie's position be true? If his opinion be correct, the carbonic acid should not leave the vessel. Let the capacity of its mouth be five times as great as that above mentioned, the gas will quit the vessel in a *few minutes*, from the greater extent of surface exposed. Now this is precisely the condition of M. Devergie's asphyxiating apartment: there is considerable extent of surface, and yet it is to be supposed according to him that the carbonic acid collects in larger proportion at the bottom of the room than it does at the top. If M. Devergie's statement were well founded, it is obvious, that in a calm state of the atmosphere the air in the streets of a crowded metropolis would be perfectly irrespirable.

In support of the erroneous view which we have just condemned, we sometimes find it stated, that carbonic acid exists in wells, on the floors of cellars and grottoes; and the Grotto del Cane near Naples, is often quoted as an instance where the gas is collected on the soil. But in all these cases it is forgotten that the source is permanent, and that the gas is continually issuing from fissures in the soil, or from decomposed matter.

To our minds, it is not proved satisfactorily that any separation of gases ever takes place, such as that above assumed; and while there is this want of evidence on the one side, there are numerous facts on the other, which render it certain that such a separation does not ensue, at least to any perceptible extent.

In the ninth query we have a practical (and in our view a most erroneous) deduction from the circumstances which we have just been considering. Thus it is assumed as probable, that of two persons in a small chamber where charcoal is burning, the one on the floor may perish, while another three or four feet above that level may escape, owing to this supposed separation of gases. We do not believe that such a case is likely to occur; and if it did take place, the result would probably depend upon whether the individuals were equally near the source of combustion. M. Devergie admits, and indeed proves, that the poisonous gas becomes equally diffused all over the room: that it may be insufficient to destroy life while thus diffused, but that it becomes destructive by slowly falling to the floor, and there forming a concentrated stratum. We have already said enough to show that if this be the case with charcoal vapour, it differs materially in its chemical and physical properties from those known to be possessed by carbonic acid. In our view, most people are killed by carbonic acid during the actual diffusion of the gas, so that the subsequent separation on cooling, even if it took place, would have very little influence in producing a fatal result. Besides, we must protest against the correctness of the inference so generally drawn at coroner's inquests in these cases, that because a person is found lying asphyxiated on a floor, he must therefore have been affected by the gas while in the recumbent posture. It is possible that he may have been sitting or standing, and have fallen asphyxiated. To analyze the stratum in which his head is found lying, and infer from the quantity of carbonic acid there discovered, that he has been attacked and has died in the recumbent posture, is absurd; since, unless the body be accidentally supported, every asphyxiated subject must be thus found.]

---

*On the Signs of Suspension, before and after Death.* By M. Orfila.

At the sitting of the Royal Academy of Medicine, October 6, 1840, M. Orfila read a long memoir on suspension. He passed in review all the characters given by different authors to determine whether suspension had taken place during life or after death. The presence of zoospermata in the urethra is not, according to M. Orfila, any proof that the subject had been suspended during life, for semen may arrive there from other causes, sometimes even in a manner purely mechanical. In two cases of suicide by suspension reported by M. Ollivier, of Angers, there was no trace of injection or congestion either of the scrotum or penis; the latter was remarkably flaccid and the corpus cavernosum very pale. In these cases death had been very rapid. M. Orfila concludes with M. Ollivier that the turgescence of the penis is a mechanical effect of position, and thinks that the state of the vertebral column is a highly important consideration. He made experiments on twenty dead bodies suspended after death at various heights from the ground, and subjected them to different degrees of traction and rotation, but in none was the atlas luxated upon the second cervical vertebra. In one case extravasated blood was found in the vertebral canal; an important fact proving, contrary to the opinion of most writers, that this character is not a proof of suspension during life. The author concludes his memoir with the following propositions:

1. None of the signs given by authors as sufficient to distinguish suspension during life and after death are to be depended upon.
2. When with the signs of asphyxia there is neither ecchymosis, furrow around the neck, fracture, nor luxation, the suspension *may* have taken place during life, but more probably after death.
3. If there is ecchymosis in the skin and cellular tissue about the furrow of the cord, it is most probable that the suspension has been during life.
4. If some of the vertebral ligaments are torn, the hyoid bone or thyroid cartilage fractured, ecchymosis about the neck, fracture of the vertebræ and no other lesions upon the body, suspension may have followed death, or may have been the result of suicide.
5. If with the signs of asphyxia any of the cervical vertebræ are broken, and there are no marks of other violence, the case is probably one of homicide.
6. If there is luxation of the atlas upon the axis we may affirm that suspension has followed death, unless there be caries of one of the vertebræ.
7. The moral and circumstantial evidence is in general more capable of proving whether the suspension has been before or after death.

*Gazette Médicale de Paris. Octobre* 10, 1840.

